# Cryoglobulinemia vasculitis associated with adult‐onset Still's disease

**DOI:** 10.1002/ccr3.8632

**Published:** 2024-03-07

**Authors:** Noriharu Nakagawa, Ai Fujii, Yoshimichi Ueda, Masahide Yamazaki

**Affiliations:** ^1^ Department of Internal Medicine Keiju Medical Center Nanao Ishikawa Japan; ^2^ Department of Nephrology Kanazawa Medical University Kahoku Ishikawa Japan; ^3^ Department of Pathology Keiju Medical Center Nanao Ishikawa Japan

**Keywords:** adult‐onset Still's disease, cryoglobulinemia, purpura, vasculitis

## Abstract

**Key Clinical Message:**

The present case indicates that cryoglobulinemia vasculitis should be considered in the differential diagnosis of purpura in patients with adult‐onset Still's disease (AOSD).

**Abstract:**

The presence of purpura is suggested in adult‐onset Still's disease (AOSD) hematological complications of hemophagocytic syndrome, disseminated intravascular coagulation, or thrombotic microangiopathy. We herein report a case of AOSD complicated by cryoglobulinemia vasculitis presenting with purpura.

A 59‐year‐old woman visited a general hospital with the chief complaint of peripheral joint swelling and edema. Laboratory findings were as follows: white blood cell (WBC) count, 6500/μL; hemoglobin (Hb), 10.9 g/dL; platelet count, 171,000/μL; aspartate aminotransferase, 111 U/L; alanine aminotransferase, 44 U/L; lactate dehydrogenase (LDH), 1040 U/L; C‐reactive protein (CRP), 4.32 mg/dL; ferritin, 6990.0 ng/mL; rheumatoid factor, negative; and cryoglobulin, negative. She visited our hospital a week later with fever. Pale erythema was observed on her back. The laboratory data showed improvement. However, her fever persisted, and several days later, purpura appeared on her lower legs (Figure 1A). Laboratory findings on the appearance of purpura were as follows: WBC count, 4960/μL; Hb, 9.9 g/dL; platelet count, 162,000/μL; LDH, 732 U/L; CRP, 13.54 mg/dL; and ferritin, 15,324.4 ng/mL. Computed tomography revealed mild enlargement of the cervical and axillary lymph nodes. Bone marrow examination revealed hemophagocytosis, and the serum cytokine profile was neopterin, 52 nmol/L (reference range: <5 nmol/L); interleukin (IL)‐18, 143,000 pg/mL (reference range: <500 pg/mL); IL‐6, 26 pg/mL (reference range: <5 pg/mL); soluble tissue necrosis factor receptor (sTNF‐R) I, 2820 pg/mL (reference range: 484–1407 pg/mL); and sTNF‐RII, 12,800 pg/mL (reference range: 829–2262 pg/mL). Lymphoma was not detected in two bone marrow examinations and random skin biopsies. Based on these findings, the patient was diagnosed with adult‐onset Still's disease (AOSD). She also had low complement levels and cryoglobulin seroconversion at the time of purpura appearance (CH50, <5 U/mL; C3, 91 mg/dL; C4, 2.1 mg/dL; cryoglobulin, positive). Skin biopsy of the purpura revealed multiple small‐vessel thromboses, vascular endothelial injury with surrounding leukocytoclast formation, and extravasation of red blood cells in the superficial dermis (Figure 1B). The thrombosis was positive for periodic acid‐Schiff (PAS) staining, and these findings were consistent with cryoglobulinemia vasculitis. After admission, the purpura spontaneously improved. Her complement levels were elevated (CH50, 47 U/mL; C3, 183 mg/dL; C4, 14.9 mg/dL), and cryoglobulin was negative before steroid administration.

**FIGURE 1:**
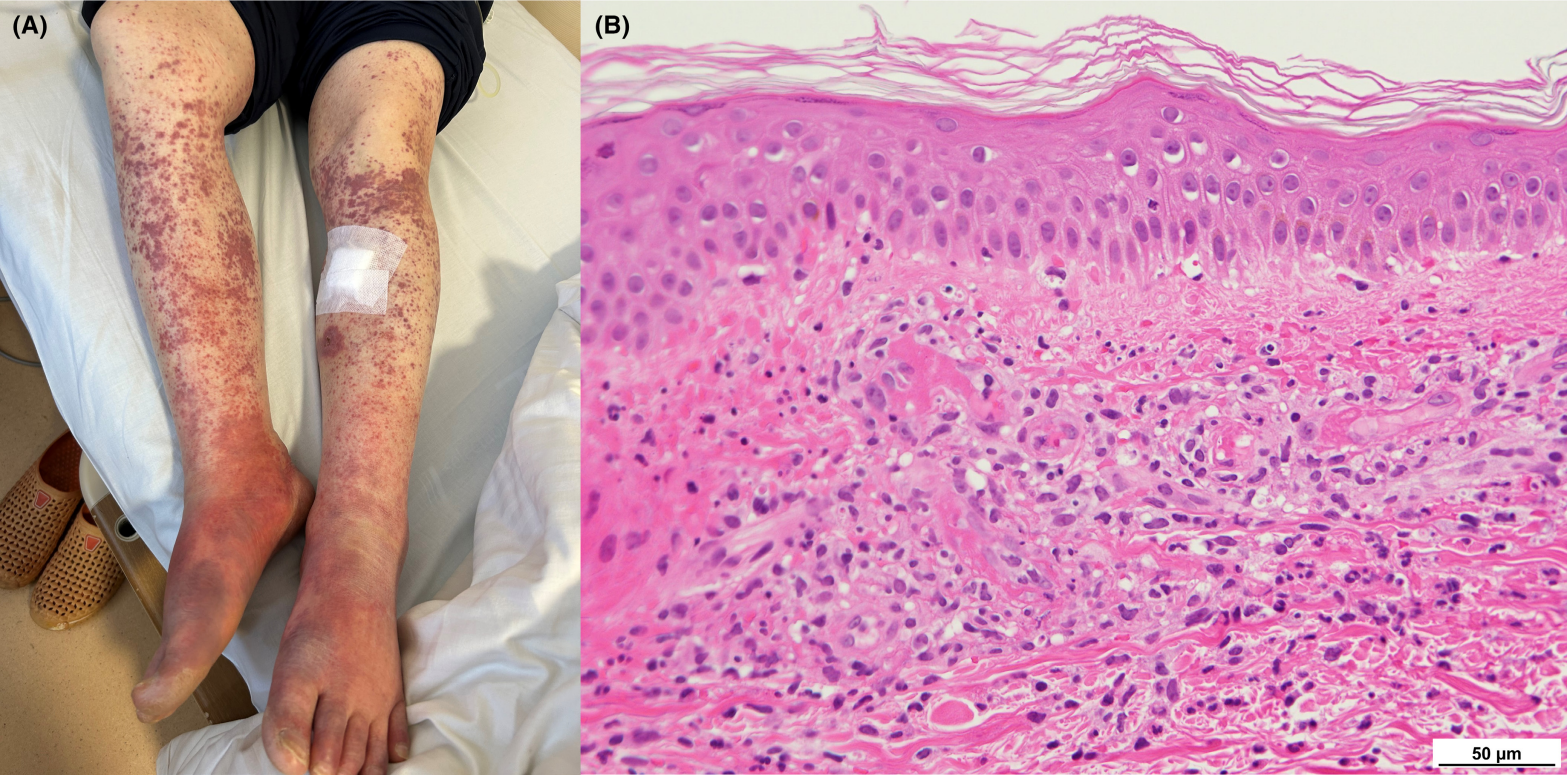
(A) The patient presented purpura on both legs. (B) A skin biopsy showed multiple small‐vessel thromboses, vascular endothelial injury with surrounding leukocytoclast formation and extravasation of red blood cells in the superficial dermis (hematoxylin–eosin).

AOSD is recognized as a systemic autoinflammatory disease, and its pathogenesis is thought to be a cytokine storm mediated by macrophages. The skin rash in AOSD is typically salmon pink and macular or maculopapular. Purpura is atypical in AOSD, while its presence is suggested in AOSD hematological complications of hemophagocytic syndrome (HPS), disseminated intravascular coagulation (DIC), or thrombotic microangiopathy (TMA).^1^ Cryoglobulinemia refers to the presence of circulating cryoglobulins and generally leads to a systemic inflammatory syndrome characterized by fatigue, arthralgia, purpura, ulcers, neuropathy, and/or glomerulonephritis.^2^ Our patient met Yamaguchi's criteria for AOSD,^3^ with fever, joint symptoms, pale erythema, lymph node swelling, liver dysfunction, and rheumatoid factor negativity. A skin biopsy revealed that the purpura on her lower legs was caused by cryoglobulinemia vasculitis.

The most frequent autoimmune disease that causes cryoglobulinemia is Sjögren's syndrome, whereas AOSD is infrequent.^4^ The present case was negative for the hepatitis C virus, which is a common underlying disease associated with cryoglobulinemia. We encountered a case of AOSD complicated by cryoglobulinemia vasculitis presenting with purpura. Based on the clinical course of this case, it is possible that some trigger manifested cryoglobulinemia in the abnormal immune activation state in AOSD. The purpura on her lower legs was a macroscopic finding that appeared to be vasculitis, and this finding may have led to the misdiagnosis of vasculitis as the cause of the fever of unknown origin. As a result, the finding of the purpura that was observed on her lower legs also appeared with AOSD thus seems to be important. In addition to HPS, DIC, and TMA, cryoglobulinemia vasculitis should be considered in the differential diagnosis of purpura in AOSD patients. Furthermore, since cryoglobulinemic vasculitis associated with AOSD may be transient, careful follow‐up is necessary in such patients.

## AUTHOR CONTRIBUTIONS

**Noriharu Nakagawa:** Conceptualization; investigation; writing – original draft. **Ai Fujii:** Investigation; writing – review and editing. **Yoshimichi Ueda:** Writing – review and editing. **Masahide Yamazaki:** Writing – review and editing.

## FUNDING INFORMATION

The authors received no specific funding for this work.

## CONFLICT OF INTEREST STATEMENT

The authors declare no conflicts of interest in association with the present study.

## CONSENT

Written informed consent was obtained from the patient. This study was approved by the Ethics Committee of Keiju Medical Center.

## Data Availability

No datasets were generated or analyzed during this case report.
